# Supplementary material for: Grand multiparity and its associated factors in Zambia: Evidence from the 2018 Zambia Demographic and Health Survey
Source: PLOS Glob Public Health. 2025 Aug 28;5(8):e0005051. doi: 10.1371/journal.pgph.0005051 (PMC12393760; doi:10.1371/journal.pgph.0005051)
Supplement: S1 Table — (DOCX) [file pgph.0005051.s001.docx]

|  |  |  |
| --- | --- | --- |
| **Variable** | **VIF** | **1/VIF** |
| Maternal education | 1.46 | 0.686 |
| Wealth index | 1.44 | 0.694 |
| Age at first birth | 1.43 | 0.697 |
| Age at first sex | 1.42 | 0.704 |
| Partner education | 1.29 | 0.776 |
| Age | 1.14 | 0.876 |
| Access to media | 1.11 | 0.899 |
| Polygamous union | 1.1 | 0.910 |
| Sex of household head | 1.07 | 0.934 |
| Region | 1.07 | 0.938 |
| Employment status | 1.04 | 0.961 |
| Contraceptive use | 1.04 | 0.962 |
| Previous birth interval | 1.01 | 0.993 |
| Mean VIF | 1.2 |  |
|  |  |  |
