# Supplementary material for: Grand multiparity and its associated factors in Zambia: Evidence from the 2018 Zambia Demographic and Health Survey
Source: PLOS Glob Public Health. 2025 Aug 28;5(8):e0005051. doi: 10.1371/journal.pgph.0005051 (PMC12393760; doi:10.1371/journal.pgph.0005051)
Supplement: Attachment — Submitted filename: Response to Reviewers.docx [file pgph.0005051.s005.docx]

The Editor, PLOS Global Public Health

16th December 2024.

Dear Editor,

Subject: Response to Reviewer Comments on Manuscript ID - PGPH-D-24-02228

I am writing to address the concerns raised by the reviewers regarding our manuscript titled ***“******Grand multiparity and its associated factors in Zambia: Evidence from the 2018 Zambia Demographic and Health Survey.”*** submitted to PLOS Global Public Health. We appreciate the feedback provided, and we have made all necessary revisions to improve the quality of the manuscript. The following is a summary of how we have addressed each concern:

Reviewer #1: The authors describe the prevalence of grand multiparity in a sample of Zambian women with a recent birth, and the associated factors.
Grand multiparity is an important but often overlooked risk factor, common in sub-Saharan Africa. It is an important analysis, as grand multiparity has mostly been studied in hospitals, thus on a subgroup of high parity women who reach hospitals for childbirth. Population-based studies are not common. I have read the analysis with interest.

Major comments -
1. The authors describe and discuss the prevalence of grand multiparity. In view that a part of the women were excluded - women under 20 and women with 0 or 1 children - the study cannot report prevalence in the country, but only in their sample. Thus the study cannot report that 39% of women in Zambia had experienced grand multiparity. 39% of women in this sample were grand multiparous. If all women had been included in the denominator, the study could report the percentage of grand multiparity in the population. As it is, researchers must report that this is the percentage in their sample. The percentage reported overestimates the percentage of grand multiparous women in the population in Zambia.
I feel that the study would improve if the researcher reported characteristics of all women (thus reporting the prevalence in the population) at the beginning, then describing analysis of the subgroup they have chosen to analyse.

Answer: **All women were included in the denominator for the percentage distributions and bivariate analysis. Our study reports that 23.83% of women in Zambia are grand multiparous. For the regression analysis, women under 20 years of age and those with fewer than two children were excluded**.

Additionally, I think women who are parity over 10 should not be excluded from the "grand multiparity" group. It is a small number and will not change analysis significantly. However, they share the same risk factors.

Answer: **Women with 5 or more children were included at all levels of analysis, as advised by the reviewer.**

2. The significance of the paper will increase if the authors examine the significance of the risk factor from a public health point of view. Specifically, the discussion will benefit by considering the public health significance of this sizable group. The authors have focused on prevention of the condition (education, FP). However, the problem of high fertility in sub-Saharan is not easy to solve, and these women and their babies exist and are at higher risk. Additionally, morbidity and mortality among these women have wide impact among their families and communities. This was described by Fathalla in "The long road to maternal death", in 1987. What can be done in communities, facilities for these women? What are the implication of this research in current maternal health care policy in Zambia?
Also, should comment on how their findings fit into the existing literature on women of high parity in sub-Saharan Africa. Recent multicountry evidence (including Zambia in the analysis) (<https://www.ncbi.nlm.nih.gov/pubmed/38262683>) indicates that GM (or high parity) is a vulnerability that adds on to poverty in rural settings. Women of high parity rarely give birth in hospitals, despite being at increased risk. For example, how does the study add to the picture of disadvantage of these women?

Answer: **Thank you for your comments. In response, we have expanded the discussion to emphasize the vulnerabilities faced by grand multiparous women in rural Zambia, highlighting the need for targeted interventions such as improved access to skilled care, community-based initiatives, and addressing barriers like transportation and financial constraints. We also stress the importance of family planning and education programs to improve birth spacing. Our study contributes to the literature by underscoring the disadvantage of grand multiparous women, particularly in rural areas, and calls for maternal health policies that prioritize this group through enhanced services and community health worker support. See page 14 and 15.**

Minor comments:

Reference 4 - The correct name of the author is Salomon

Answer: **This has been corrected to the correct name. Please refer to page 3.**

In the introduction, authors should note that Few studies have examined grand multiparity from a population perspective. Most studies are based on hospital populations

Answer: **It has been noted that few studies have utilized nationally representative data, with most relying on hospital-based data. Please refer to page 3**.

Low-resource/high resource settings are preferable terms compared to developed/developing

Answer: **This has been changed to "low- or high-resource settings.**

Methods
Authors should explain why variable wealth has been categorized into poor, middle, rich, rather than into quintiles

Answer: **Studies have categorized wealth into three groups by combining the "poorest" and "poorer" categories and the "richer" and "richest" categories. To ensure sufficient sample sizes in each category for robust analysis, as done in previous studies
 - (Adewuyi et al., 2017; Dasa et al., 2022) . This approach also enhances comparability with similar research utilizing nationally representative datasets. See page 5**

Adewuyi, E. O., Zhao, Y., Auta, A., & Lamichhane, R. (2017). Prevalence and factors associated with non-utilization of healthcare facility for childbirth in rural and urban Nigeria: Analysis of a national population-based survey. *Scandinavian Journal of Public Health*, *45*(6), 675–682. https://doi.org/10.1177/1403494817705562

Dasa, T. T., Okunlola, M. A., & Dessie, Y. (2022). Multilevel analysis of grand multiparity: Trend and its determinants in the Sidama National Regional State of Ethiopia: a cross-sectional study design from demographic and health survey 2000-2016. *BMJ Open*, *12*(8), 1–11. https://doi.org/10.1136/bmjopen-2022-061697

Title Table 3 – Bivariate analysis of women’s parity (low parity and grand multiparity) by background characteristics

**Answer: The table has been renamed see page 9**

Results
“Maternal education was strongly associated with grand multiparity”. This sentence should be reworded to maternal education showed a strong negative association with GM

**Answer: This has been revised – see page** 13

Discussion - see above

We believe these revisions have strengthened our manuscript, and we are confident that it now meets the high standards of PLOS Global Public Health. Enclosed with this letter, please find the revised manuscript along with a detailed point-by-point response to the reviewers’ comments. Thank you for your consideration. We look forward to your favorable review and are hopeful for a positive outcome.

Yours sincerely,

Peter Mumba – Lead author
