# Supplementary material for: Grand multiparity and its associated factors in Zambia: Evidence from the 2018 Zambia Demographic and Health Survey
Source: PLOS Glob Public Health. 2025 Aug 28;5(8):e0005051. doi: 10.1371/journal.pgph.0005051 (PMC12393760; doi:10.1371/journal.pgph.0005051)
Supplement: Attachment — Submitted filename: PGHS-D-24-02228_R1_3.docx [file pgph.0005051.s004.docx]

**Grand multiparity and its associated factors in Zambia: Evidence from the 2018 Zambia Demographic and Health Survey.**

**PGPH-D-24-02228R1**

1. Recommendation (Answer options: Accept, Minor Revision, Major Revision, Reject)

- Rejected
2. Is the manuscript technically sound, and do the data support the conclusions? (Answer options: Yes, No, Partly)

-The data partly support the conclusion
3. Has the statistical analysis been performed appropriately and rigorously? (Answer options: Yes, No, I don’t know, N/A)

-Yes
4. Does the manuscript adhere to the PLOS Data Policy? Additional details can be found at <http://journals.plos.org/plosone/s/materials-and-software-sharing>. (Answer options: Yes, No)

-No. No new information is here.
5. Is the manuscript presented in an intelligible fashion and written in standard English? (Answer options: Yes, No)

-No
6. Review Comments to the Author (minimum 200 characters)

-In fact, ZDHS 2024 was published. I cannot found any indication to publish this article that analyze ZDHS 2018 data.
7. PLOS authors have the option to publish the peer review history of their article (<https://journals.plos.org/plosone/s/editorial-and-peer-review-process#loc-peer-review-history>). If published, this will include your full peer review and any attached files. Do you want your identity to be public for this peer review? (Answer options: Yes, No)

-Yes
7a. If you answered "Yes" above, that you would like your identity to be public, please provide your full name, as it should appear on the published peer review. Please do not sign the review on behalf of another person.

-Dr. Khursheda Akhtar

Confidential to Editor:
1. Do you have any potentially competing interests? If none, type "None." Our policy on competing interests can be found at <http://journals.plos.org/plosone/s/competing-interests>.

-None
2. Did you receive any assistance in preparing this review (e.g. from a post-doc or graduate student)? If yes, please provide their name and title. If you have chosen to reveal your identity to the authors in question 7 above, please include the name of the person who assisted you in the response to question 7 as well. (optional)

-No
3. Do you want to receive recognition for this review on a Web of Science researcher profile? (<https://clarivate.com/products/scientific-and-academic-research/research-discovery-and-workflow-solutions/researcher-profiles/>)? If you opt in, your Web of Science profile will automatically be updated to show a verified record of this review in full compliance with the journal's review policy. If you don't have a Web of Science profile, you will need to first create a free account. (Answer options: Yes, No)

-Yes
