# Supplementary material for: Grand multiparity and its associated factors in Zambia: Evidence from the 2018 Zambia Demographic and Health Survey
Source: PLOS Glob Public Health. 2025 Aug 28;5(8):e0005051. doi: 10.1371/journal.pgph.0005051 (PMC12393760; doi:10.1371/journal.pgph.0005051)
Supplement: Attachment — Submitted filename: Response to Reviewers Comments.docx [file pgph.0005051.s007.docx]

The Editor, PLOS Global Public Health

02 February, 2024.

Dear Editor,

Subject: Response to Reviewer Comments on Manuscript ID - PGPH-D-24-02228

I am writing to address the concerns raised by the reviewers regarding our manuscript titled ***“******Grand multiparity and its associated factors in Zambia: Evidence from the 2018 Zambia Demographic and Health Survey.”*** submitted to PLOS Global Public Health. We appreciate the feedback provided, and we have made all necessary revisions to improve the quality of the manuscript. The following is a summary of how we have addressed each concern:

**Reviewer 1**

Overall, the authors have addressed the concerns contained in the previous review. The manuscript has gained in clarity and now findings are derived from the statistical analysis.

Answer: **Thank you for your comments, we really appreciate your inciteful feedback.**
I recommend the following minor changes:
1. Abstract

First sentence
There are socio-economic determinants to high fertility, ( poverty and low women’s education), rather than just lack of access to contraception/medical care. The sentence should be reworded to ( …) high resources setting, partly due to …

Answer: **Thank you for your comment – this has been revised. See page 2**

2. Methods
Third paragraph/page 4
Text refers to Table 5, but I cannot find it.
Also, authors report that there was no collinearity between independent variables, but then that marital status was dropped due to multicollinearity.
Please address this.

Answer: **This has been revised. See page 5**,
3. Results
Table 2 – 1 decimal for percentages is sufficient

Answer: **This has been revised as suggested by the reviewer**

Table 4 – as the authors have correctly reported p-values, asterisks are not necessary and should be removed to make the table more easily readable.

Answer: **This has been revised.**
4. Discussion – I suggest adding a first paragraph summarizing your main findings.

Eg this study analysed over 13000 women to study prevalence and risk factors associated with grand multiparity in Zambia. We found approximately one quarter of women in Zambia were grand multipara. Using multivariate logistic regression, we found age, length of marriage duration, poverty, etc were associated to grand multiparity.

The discussion of the findings should then follow.

Answer: **Thank you for comment: This has been addressed see page 13**

5. Conclusion – I suggest to move the conclusion to the end. It should summarize findings and relevant policy recommendations. The first sentence reporting risks associated to grand multiparity from existing literature should not be part of the conclusion.

Answer: **Thank you for your comment. This has been addressed see page 17.**

We believe these revisions have strengthened our manuscript, and we are confident that it now meets the high standards of PLOS Global Public Health. Enclosed with this letter, please find the revised manuscript along with a detailed point-by-point response to the reviewers’ comments. Thank you for your consideration. We look forward to your favorable review and are hopeful for a positive outcome.

Yours sincerely,

Peter Mumba – Lead author
