# Supplementary material for: Grand multiparity and its associated factors in Zambia: Evidence from the 2018 Zambia Demographic and Health Survey
Source: PLOS Glob Public Health. 2025 Aug 28;5(8):e0005051. doi: 10.1371/journal.pgph.0005051 (PMC12393760; doi:10.1371/journal.pgph.0005051)
Supplement: Attachment — Submitted filename: Comments for PLOS Global public health.docx [file pgph.0005051.s006.docx]

**Comments for the authors**

**Title: Grand multiparity and its associated factors in Zambia: Evidence from the 2018 Zambia. Demographic and Health Survey.** From the beginning knowing prevalence of grand multiparity is not that much interesting rather it was better to include the outcomes of grand multiparity.

**Abstract**; page 2, the definition of grand multiparity should place at the beginning of the paragraph, and the abstract must be subtitled as background, methods, result, conclusion, and keywords. At the above you said the study population were women aged between15-49, but some lines below you exclude age less than 20, why?

You also said women having less than two children were excluded, this is known by default.

**Results**: page 8, include the confidence interval of your result.

Generally you have no new things added, by default your significant factors are known and familiar, you can get from different obstetrics books, so what new thigs you add rather than known factors? This concept disagree with goal of research. It was better, if you were study the prevalence with maternal as well as fetal outcomes of grand multiparity.
