# Supplementary material for: Grand multiparity and its associated factors in Zambia: Evidence from the 2018 Zambia Demographic and Health Survey
Source: PLOS Glob Public Health. 2025 Aug 28;5(8):e0005051. doi: 10.1371/journal.pgph.0005051 (PMC12393760; doi:10.1371/journal.pgph.0005051)
Supplement: Attachment — Submitted filename: Response_to_Reviewers_auresp_3.docx [file pgph.0005051.s008.docx]

The Editor, PLOS Global Public Health

18 May, 2025.

Dear Editor,

Subject: Response to Reviewer Comments on Manuscript ID - PGPH-D-24-02228

I am writing to address the concerns raised by the reviewers regarding our manuscript titled ***“******Grand multiparity and its associated factors in Zambia: Evidence from the 2018 Zambia Demographic and Health Survey.”*** submitted to PLOS Global Public Health. We appreciate the feedback provided, and we have made all necessary revisions to improve the quality of the manuscript. The following is a summary of how we have addressed each concern:

**Reviewer 3**

Title: Grand multiparity and its associated factors in Zambia: Evidence from the 2018

Zambia. Demographic and Health Survey. From the beginning knowing prevalence of grand multiparity is not that much interesting rather it was better to include the outcomes of grand multiparity.

Answer: **Thank you for your comment. We added that as recommendation for future research.**

Abstract; page 2, the definition of grand multiparity should place at the beginning of the paragraph, and the abstract must be subtitled as background, methods, result, conclusion, and keywords. At the above you said the study population were women aged between15-49, but some lines below you exclude age less than 20, why?

Answer: **Thank you for your comments the abstract has been structed as suggested by the reviewer and the women less than 20 have been included in the analysis in the multivariate analysis. We dropped them because they had lower risks of being multipara. See page 1**

Results: page 8, include the confidence interval of your result.

Answer: **The confidence intervals have been included**

Reviewer #3: Generally you have no new things added, by default your significant factors are known and familiar, you can get from different obstetrics books, so what new thigs you add rather than known factors? This concept disagree with goal of research. It was better, if you were study the prevalence with maternal as well as fetal outcomes of grand multiparity.

Answer: **Thank you for your comment. While we acknowledge that several of the factors associated with grand multiparity are well-documented in obstetric literature, our study provides a novel contribution by examining these factors using nationally representative data from Zambia—a context where such evidence is scarce. To the best of our knowledge, only one study in Africa has used a similar dataset, while most existing studies rely on hospital-based data, which may not be generalizable to the broader population. Our analysis thus addresses this gap by offering a more comprehensive and population-level understanding of the determinants and implications of grand multiparity. We believe this approach aligns with the research objective and offers valuable insights for public health planning and maternal care strategies.**

Reviewer #4: The paper is interesting and provides valuable insights into the prevalence and related factors of grand multiparity in Zambia. It highlights a critical topic that should be properly addressed to reduce high fertility rates mainly in low-income countries. In general, the document is rich and well structured. Yet, the below comments would help improve its quality.

Abstract:
It is not easy to read mainly for the statistic part. Simplify and provide clear sentences.

Answer: **This has been revised – see page 1**

Background:
This part is comprehensive, but it is important to provide some statistics concerning the prevalence of grand multiparity.
Provide some details about the context and the way it may affect the fertility rate.
Highlight the initiatives taken by the instances to reduce fertility rate. Ok I found this explanation in the discussion unless you want to reflect on it briefly in the background.

Answer: **Thank for you comment and this has been revised**

Methods
I did not understand why you excluded women of less than 20. In sub-Saharan African, including Zambia, women may become sexually active at a very early age and are more exposed to multiparity. This is revealed just at the beginning of the results where you mentioned that “more than two-thirds (68%) of these women had their first childbirth before the age of 20, and the majority (91%) initiated sexual activity before turning 20.”

Answer: **Thank you for your comment – we included the women aged less that 20. We thought because they were less likely to be grand multipara. We do agree with your comment and added them to the analysis.**

p. 8: Age at first birth <20, 20-34, 25+. 34 should be 24.

Answer: **This has been revised.**

Answer: **Thank for your observation. This has been revised.**
Accessibility to healthcare services is a confounding variable that is not included in the table and ought to be mentioned in the limitations unless you add it in the analysis.

Answer: **This has been added by making a composite variable of 5 variables. See page 6**

Results
What percentage of women were in the younger age groups (15-19, 20-24)?
Arrange the % in table 2
Polygamous marriage (number of other wives)
No 4532(67.46%) 2186(32.54%)
Yes 389(45.89%) 459(55411%)

Answer: **This has been revised**
Does partners’ education affect grand parity? Why this was excluded from the analysis?

Answer: **This has been included in the bivariate analysis and multivariate analysis. See page 11 and 14**

Discussion
Women who used contraceptives were 29% more likely to experience grand multiparity compared to non-users. This could be interpreted the way you did but it might be that women are not using the contraceptive methods properly because of low education and poor life conditions with no access to media information. They might also be using contraceptive methods that are not so reliable.

Answer: **Thank you for your comment. This has been reflected in our discussion. See page 15**
How do you explain the below results:

Women with a previous pregnancy interval of 24 months or more had more than twice the odds of grand multiparity compared to those with shorter intervals (aOR=2.76, 95% CI: 1.764-4.321).
This requires explanation and justification, as usually the opposite is true.

Answer: **This has been corrected. Its the other way round as it was reported in the discussion that women with shorter birth intervals are more likely to be grand multiparous.**
Discuss the potential influence of polygamy on reproductive patterns as this is present among 11% of the participants.

Answer: **This has been discussed – see page 16**

It might be also interesting to further discuss why the implemented policies did not properly meet their aims. This could be related to the healthcare infrastructure, limited number and unskillful human resources, accessibility and adaptability of service, lack of culturally sensitive services, etc.

Answer: Thank you for your comment. This has been added to the discussion – see page 18

Conclusion
Add study limitations.

Answer: **Limitations and strengths of the study have been added**
Recommendations to improve reproductive health services to reduce grand multiparity focusing on the gaps in healthcare services and the need to equip healthcare providers to address this issue more efficiently.
Answer: **This has been revised. See page 19**

We believe these revisions have strengthened our manuscript, and we are confident that it now meets the high standards of PLOS Global Public Health. Enclosed with this letter, please find the revised manuscript along with a detailed point-by-point response to the reviewers’ comments. Thank you for your consideration. We look forward to your favorable review and are hopeful for a positive outcome.

Yours sincerely,

Peter Mumba – Lead author
